# Autism, bullying, and mental health: a comprehensive systematic review

**DOI:** 10.3389/fpsyt.2025.1653663

**Published:** 2025-12-05

**Authors:** Wid H. Daghustani, Eid G. Abo Hamza, Rachel Hogg, Ahmed Moustafa

**Affiliations:** 1 Special Education Department, Learning and Developmental Disabilities Programme, Arabian Gulf University, Manama, Bahrain; 2 College of Arts, Humanities and Social Sciences, University of Sharjah, Sharjah, United Arab Emirates; 3 Tanta University, Tanta, Egypt; 4 School of Psychology, Charles Sturt University, Albury, NSW, Australia; 5 Department of Human Anatomy and Physiology, University of Johannesburg, South Africa; 6 University, Gold Coast, QLD, Australia

**Keywords:** autism, vulnerability, bullying, risk factors, prevalence, mental health, prevention, protection

## Abstract

This review investigates the relationship between autism, vulnerability to bullying, and the impact of bullying on mental health for this cohort. Neurotypical social mores can be exclusionary, creating social risks for autistic people, while differences in communication and social engagement can impact peer-to-peer interaction, making autistic people more vulnerable to bullying and social discrimination. The current systematic review investigates risk factors related to the bullying of autistic people, considering both societal and individual factors. Our PRIMA guided search reported 74 studies. Our results show that the prevalence of bullying of autistic individuals vary across studies, autistic individuals face verbal, social, and physical bullying, and that bullying was found to lead to the development of depression, anxiety, and social withdrawal. The importance of education, creating inclusive environments, building resilience, collaborative efforts, policy and legislation, mental health support, and prevention and protection implications, are discussed. By implementing these strategies, we can work to reduce vulnerability to bullying in autistic people as well as promote their overall well-being. This review emphasises the importance of comprehensive interventions and support systems in combating bullying and improving the lives of autistic people.

## Introduction

Autism is a condition that affects communication, social interaction, behaviour, and peer relationships (1, 2). These differences, alongside non-neuro inclusive societal social and interpersonal expectations, mean that autistic individuals face unique challenges when it comes to social interaction, which in turn make autistic people more vulnerable to bullying and social discrimination (3). Autistic individuals may express unique social behaviours that do not follow conventional social norms and these behaviours may be perceived negatively, in part because of their unfamiliarity given the ways in which difference may commonly be interpreted as threat. Such differences may result in miscommunication regarding voice tone and body language, increasing vulnerability to mockery and ridicule (4). This issue may be critical in the mental health outcomes of those who identify as autistic or have a diagnosis of autism spectrum disorder. Indeed, research suggests bullying can have a devastating effect on individuals with autism, leading to increased anxiety, depression, and social withdrawal (5, 6), in addition to negative impacts on academic and social functioning (7), as will be explored in this review.

Experiences of bullying and discrimination are also consequential for masking behaviours, where autistic individuals mimic neurotypical social and other behaviours as a protective mechanism designed to facilitate conformity to dominant societal norms (8, 9). Masking behaviours commonly represent a survival strategy for autistic people but also create a significant cognitive and psychosocial load (8) and are also implicated in experiences of psychological trauma for autistic people (10).

Bullying is understood as a systematic repetitive behaviour or set of behaviours, performed with the intention of causing emotional and physical harm and may be verbal, social, or physical in nature. Such intentional, repetitive behaviours are aimed at harming another person physically and/or psychologically, while bullying usually occurs in the context of a power imbalance between the bully and the victim (11, 12). As Gladden et al. (13, p. 7) note, such “behaviour … may inflict harm or distress on the targeted youth including physical, psychological, social, or educational harm.”

Given societal shifts towards online modes of social interaction, bullying occurs now more in isolation and is prevalent on- and off-line, with cyberbullying a ubiquitous experience for many young people. Data indicates that 44% of young people in Australia have experienced bullying (14), while data from the United States reflects similar rates, with those aged 12–15 years of age most likely to experience online bullying. As Fidazzo (15) highlights, definitions of bullying now encompass less direct, more covert behaviours, including hostile facial expressions, gestures, and gossip. Research from the Cyberbullying Research Center (16), indicates that receiving and sharing explicit images are two key categories of cyberbullying, highlighting the increasingly sexualised nature of online bullying. Cyberbullying has a snowball effect, since one online attack can be spread throughout the web in various locations, which has a distressing effect on the victim (17, 18).

This systematic review will address the prevalence and types of bullying that occur in the context of autism, while also offering a seminal contribution to the literature in considering cultural variability in context to bullying and autism, acknowledging the ways in which the deficit model of autism influences bullying, and research on autism and bullying, across cultural contexts. Much of the research on autism and bullying has been conducted through a Western, Anglo-centric lens, while few studies have been conducted in the Arab World. This is consequential because, as Sulaimani and Daghustani (19) note, the medical model of autism remains dominant in countries such as Saudi Arabia. They describe how media depictions of autism position autism as “malfunction”, an ailment to be cured, and ultimately, a personal tragedy that limits individual potential and carries with it “shame and disappointment” (p. 2).

According to Gladden et al. (13), there are four types of bullying: verbal such as teasing and threats, physical such as hitting and spitting, relational as in social exclusion and cause damage to belongings such as books and bags. Victims or bullied are the individuals who experience the unwanted behaviour of bullying, when bullies or perpetrators are the ones who target others with the aggressive behaviours (20). **Table 1** shows a description of the types of bullying in autism.

**Table 1:** Type of bullying in autism.

	Type of bullying	Description	Reported prevalence
1	Verbal	Threats, name calling and teasing	58% Sreckovic et al. (21); 33% Maiano et al. (22)
2	Physical	physical attack, hitting and spitting	30% Sreckovic et al. (21); 50% Maiano et al. (22)
3	Relational/social	social exclusion, spreading malicious rumours and damage to belongings such as books and bags	36% Sreckovic et al. (21); 31% Maiano et al. (22)
4	Cyberbullying	online bullying	15% Sreckovic et al. (21)

In this systematic review, we will also examine risk factors for bullying, mental health outcomes associated with bullying in the context of autism, as well as implications for prevention and protection of autistic people. Finally societal and social frameworks, alongside contextual factors, are critical in creating a framework through which harmful interpersonal interactions may occur, often between neurodiverse and neurotypical individuals and these are explored in our review.

Given psychological literature on autism has tended to focus on the individual, it is easy to conceptualise the bullying of autistic people in relation to differences in communication and interests, as well as other autistic traits, furthering a deficit model of thinking that situates “the problem” and solution to bullying with the autistic person. Such thinking directs focus away from the social and psychological challenges and “deficits” of the bullying perpetrator and reinforces neurotypical behaviour as the “standard”. While it is necessary and important to conceptualise the individual differences that create social vulnerability for autistic people, as explored in our review, bullying in context to neurodivergence must also be understood in context to the purpose of bullying and the ways in which those who diverge are often excluded and socially punished, not because of their deficit, but because of their difference and the threat this is seen to represent to the dominant social class.

We seek to explore bullying here as a product of the person-environment nexus, especially where the divide between the neurotypical and the neurodiverse is crossed, rather than conceptualise bullying as a reflection of presumed deficits in the behaviour of autistic people.

Finally, terminological differences in the language used to describe autism and those who experience it have created significant division in the field. Where possible, we use identity-first language, that is, “autistic person”, to refer to the cohort under consideration. In some instances, in order to accurately represent the conceptualisation of autism reflected in the research under review, person-first language has been used.

The rationale of this comprehensive systematic review is to examine the relationship between autism and bullying with a specific focus on the mental health outcomes of autistic individuals. In particular, the review seeks to address the following question: What are the prevalence, types, risk factors, and mental health consequences of bullying among autistic individuals, and what implications can be drawn for prevention and protection? By formulating the review around this central question, the methodology and findings are anchored to a clearly defined aim, thereby enhancing systematic rigor and clarity.

## Methods

In the current systematic review, we investigated factors related to bullying of individuals with autism. Accordingly, we searched the following databases Pubmed, Google Scholar, EBSCO, and Psychinfo. The search was for any research studies till 22^nd^ of December, 2024. Our approach follows the PRISMA 2020 guidelines (23; see **Supplementary Table S1** in Supplementary Material). Screening of articles (n= 1123) was conducted by AAM and a senior research associate, and in case of disagreement, another research associate resolved these issues. To be considered for inclusion, study must be peer-reviewed.

Our inclusion criteria were as follows: participants are diagnosed with autism and main variable of study is bullying. As for search terms, we used the following combinations of three terms from three different groups: Group 1 which included terms such as autism, autistic, autism spectrum conditions, OR autism spectrum disorder AND Group 2 which included terms such as bullying, intimidation, OR victimisation AND group 3 which included terms such as mental health, prevalence, risk factors, prevention, intervention, protection, OR treatment. Importantly, this search statement slightly differed between databases due to suggested keywords or subject headings within each database. Search in all databases used in the current study revealed 1123 studies. After removing duplicates, reviews and non-peer reviews articles, 445 articles remained. We have screened titles and abstract of these articles. Two reviewers independently screened titles and abstracts, followed by full-text screening against the inclusion criteria. Any discrepancies were resolved through discussion, with a third reviewer consulted if consensus could not be reached. Data extraction was also conducted independently by two reviewers to ensure accuracy and reduce bias. Additionally, a quality assessment of each study was undertaken, considering methodological soundness, sample size, and clarity of reported outcomes. It should also be noted that the inclusion of Google Scholar alongside specialised academic databases (PubMed, EBSCO, PsychINFO) may have introduced variability in source quality, as Google Scholar indexes both peer-reviewed and non-peer-reviewed material. To mitigate this, we applied stricter screening and exclusion criteria to studies identified through Google Scholar, ensuring that only peer-reviewed and methodologically sound publications were retained.

**Figure 1:**
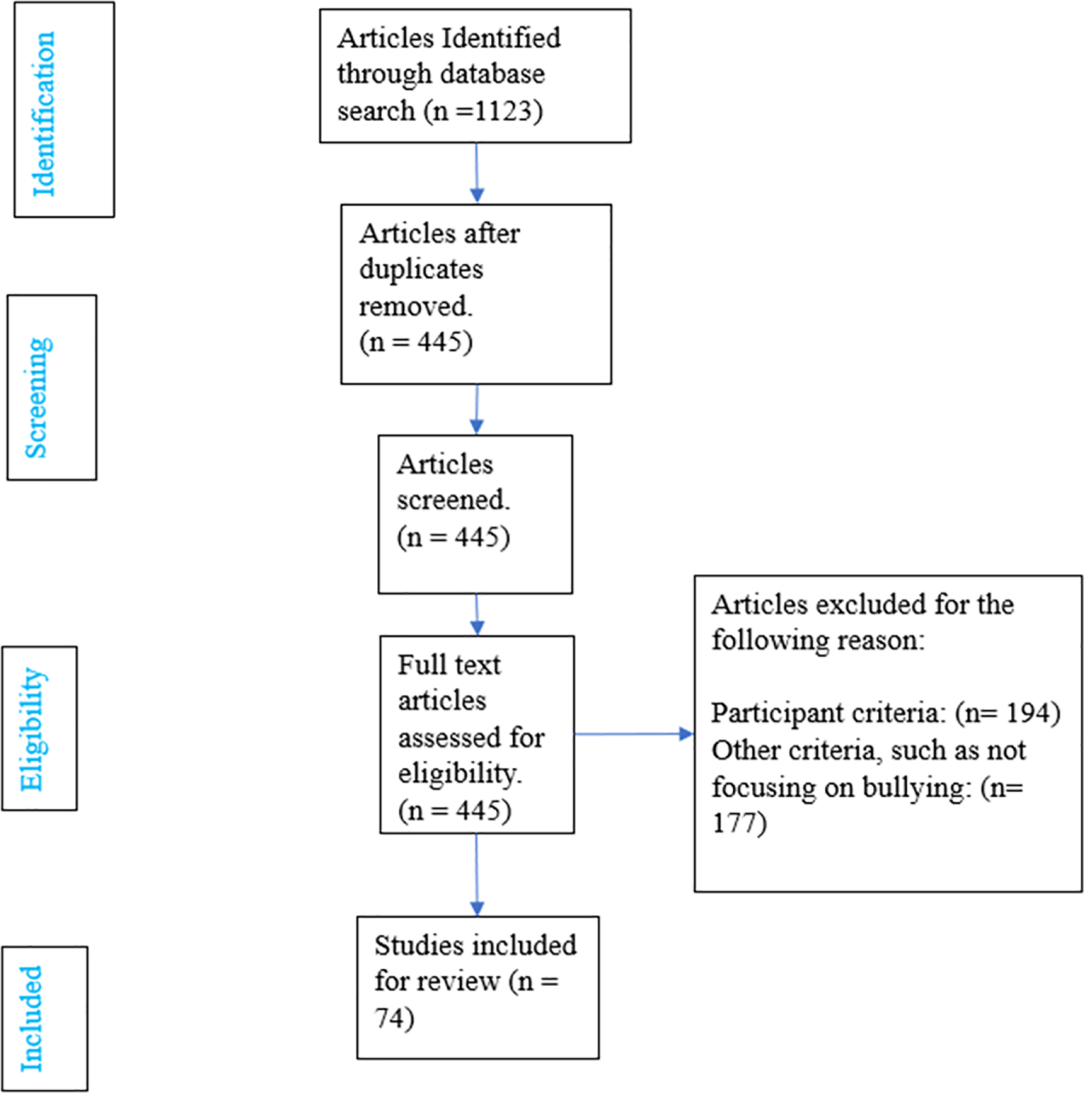
A PRISMA flowchart of our search strategy in the current systematic review.

Based on inclusion criteria, we ended up with 74 articles. The flow chat of our search method is show in **Figure 1**.

We have categorised our articles into five categories: the prevalence of bullying in autism (23 studies in total), bullying and autism in non-western context (9 studies in total), the types of bullying in autism (8 studies in total), risk factors underlying bullying in autism (15 studies in total), and the impact of bullying on the mental health of individuals with autism (21 studies in total). Below, we discuss relevant studies in each of these topics in detail. All studies found through our PRIMA research are shown in Supplementary Material (**Supplementary Table S2**). Please, note that some studies have addressed more than one of the 5 categories mentioned here. Column titled “Study Focus” in **Supplementary Table S2** mention the categories of the paper found by our PRISMA search methods.

## Results

### The prevalence of bullying in autism

Autistic individuals are more vulnerable to bullying victimisation. Research indicates they are three times more likely to experience bullying compared to their neurotypical peers (24–26). Twyman et al. (27) found that autistic individuals experienced victimisation more than non-autistic kids. Similar findings were also reported by Blake et al. (28). Different studies have reported different prevalence patterns of bullying in autism, reaching approximately 90% (29). Humphrey and Hebron (29) found that 71% of the autistic participants were being bullied when compared to 14% of the typically developing control group. However, Vân Roekel et al. (30) found that the prevalence of bullying in autistic individuals is between 6 and 46%. Other studies have reported different rates of bullying victimisation in autism ranging from 40% (31), to over 60% (32–35), or even over 90% (32, 36).

Although the variations among these different prevalence rates are not explored in the literature with the rates vary dramatically across studies (ranging from approximately 20% to over 90%), these discrepancies warrant closer consideration. Autistic individuals experience a higher rate of bullying when compared to their non-autistic peers in addition to peer rejection (37). Along these lines, Zablotsky et al. (38), surveyed 1211 parents of autistic individuals and found that autistic students are at a great risk of being victims of bullying, especially those who attend mainstreaming schools and/or present as highly neurodivergent. In addition, research has shown that bullying can lead to school refusal (i.e., not going to school) in typically developing children as well as autistic individuals (39–41).

Similarly, a study by Ball and Zhu (42) examined the prevalence of bullying behaviour among autistic and non-autistic adolescents between the age of 12–17 and found that autistic adolescents were significantly more likely to experience bullying victimisation. Similar findings were reported by Humphrey and Symes (43). Thus, bullying is a significant issue among autistic individuals in which they experience a greater risk of being exposed to bullying victimisation compared to their peers (44); however, the same study found that controlling for comorbid psychopathology in autistic individuals cancels this effect. According to Rowley et al. (4), 75% of autistic students are subjected to bullying in inclusive schools, and 40% of those who were victimised felt rejected as a result of the bullying experience. One study also found that autistic women, who are three times more likely to experience coercive sexual victimisation than non-autistic women (45).

Parental support may be a key mitigating factor in the bullying of autistic individuals. Where parents are less involved in child outcomes, vulnerability to bullying for autistic children appears to increase (46). Bullying experiences may cross the developmental trajectory from childhood into adulthood for autistic individuals, and the individual and cumulative effects of this warrant consideration. Across contexts and developmental stages, social support may be influential in determining how experiences of bullying impact autistic individuals. A study by Pryke-Hobbes et al. (47) examined the experiences of autistic adults who had been bullied in the workplace. The Pryke-Hobbes et al. (47) study found that bullying was a common experience among them and that it had a significant impact on their mental health and job satisfaction. The Pryke-Hobbes et al. (47) study also found that many autistic adults did not feel comfortable disclosing their diagnosis to their employers, which made it more difficult to address the bullying. It has also been reported that autistic adults are at a higher risk of being victim of cyberbullying than non-autistic adults (48). Like children with autism, adolescents and adults with autism are victims of different forms of bullying, including social, physical, and cyberbullying. Further, one study has also reported that autistic individuals often face bullying by their own siblings, which impact their self-esteem (49).

### Bullying and autism in a non-Western context

The relationship between bullying and disabilities in non-Western contexts have been explored in several studies. In Kuwait, Almarzouq et al. (50), explored bullying behaviour towards disabled students in mainstream schools and found that bullying is considered one of the biggest barriers in inclusive education and can lead to social isolation, decreased participation in school activities and friendship. Similar results were also reported in Jordan (51). Al-Saleh (52) interviewed four teachers in Saudi Arabia to investigate their perceptions regarding the inclusion of students with autism in schools. Al-Saleh (52) found bullying was a main factor impacting inclusive education in mainstream school settings. In Saudi Arabia, Alatawi (53) also found that Autistic individuals face sexual, social, verbal, and physical bullying as well as cyberbullying.

Similarly, Daghustani (54), reported that 69 autistic children were subjected to bullying in Bahrain, starting with verbal bullying, followed by social bullying, and finally physical bullying. Alzaidi (55) reported the same findings in their exploratory study of inclusion of autistic students in Saudi Arabia where autistic individuals had to be accompanied by a teacher during their break to protect them from bullying. Binhayyan (56) reached the same results and reported bullying as a common experience among autistic individuals who attends mainstream schools in Saudi Arabia. 17 mothers were interviewed about educating their autistic sons and reported that their children are subjected to verbal and physical bullying in the mainstream schools (57). Such research provides important insights into the prevalence and factors associated with school bullying among autistic individuals in Arab countries.

### The types of bullying in autism

Bullying may take a direct form, such as a face-to-face physical attack and/or verbal threats and name calling or indirect such as spreading malicious rumours and incitement to social exclusion (5, 58), in addition to online or cyberbullying. Autistic individuals often face different forms of bullying (7, 59). Autistic individuals are often vulnerable to bullying victimisation because of the ways in which difference may be targeted by those who seek to monitor and control what is considered socially normative behaviour. Autistic individuals are often subjected to different types of bullying such as verbal, physical and social. According to a study by Zeedyk et al. (60), autistic individuals are more likely to experience verbal and physical bullying, but less likely to experience relational or cyberbullying. However, Kloosterman et al. (61) found that autistic individuals are more likely to experience social bullying than non-autistic individuals. Research based on face-to-face interviews with parents of autistic children indicates that autistic individuals face verbal and emotional bullying (62).

Saigh and Bagadood (5) interviewed seven mothers of autistic children about their child’s experience with bullying. In addition, they asked them to fill out a questionnaire to measure the type and the frequency of bullying their child experienced. They found that the vast majority of children were teased, socially excluded and had rumours spread about them; in addition to being frightened, tricked into unwanted situations and having their possessions stolen.

In sum, as discussed here, there are different types of bullying, including verbal, physical, relational and social bullying. Most studies found that autistic people suffer from social bullying. However, it is not uncommon that children with autism are also subjected to other forms of bullying.

### Risk factors underlying bullying in autism

Factors contribute to bullying autistic individuals such as lack of understanding of their social barriers, stereotypes and negative beliefs held by others which result in discrimination and prejudice (63, 64). The severity of autism may also increase the likelihood of peer rejections and social exclusion (4). Psychosocial problems and communication issues, difficulties in interpreting social cues, specific interests, self-expression, struggles with friendships and aggressive behaviour contribute to bullying (65–67). Issues with empathy, impulsivity, understanding others, sensory processing problems and difficulties with social relationships increase the likelihood of mistreatment (68). Morales-Hidalgo et al. (69) found that bullying was associated with severity of restrictive, repetitive, behavioural and emotional problems.

The experience of discrimination and bullying is common among minorities and marginalised groups, with autism considered a condition associated with social vulnerability. One study found that certain traits in autistic individuals such as loneliness, clumsiness, and social isolation, are more associated with bullying victimisation (70). The physical expression of the autism traits such as lack of eye contact and indifferent facial expressions; alongside repetitive behaviours such as finger flicking and hand flapping are more likely contribute to discrimination and prejudice (71). In addition to the unique interests and behaviours exhibited by autistic individuals which appear socially inappropriate can also make them an easy target for bullying (1). Poor understanding of the condition that leads to stereotypes and unfavourable labelling also increase the risk of bullying victimisation and the experience of bullying at a significantly elevated rate (1).

Rodriguez et al. (72) conducted a study exploring the relationship between bullying victimisation and mental health problems in children with autism during middle to older childhood. The study found that prior mental health problems and severity of autism are related to an increase in bullying. Along these lines, Montes et al. (73) found that comorbid ADHD increases the likelihood of bullying in autistic individuals.

One study found that diagnosis with other clinical disorders is related to an increase in bullying victimisation among children with autism (74). When non-autistic individuals interact with autistic individuals this may create positive attitude and promotes acceptance, and reduce bullying (75). Children who are often victimised may have few friends to none, which leads to low social status and marginalisation. In another study, Libster et al. (37) explored the impact of gender and social skills on bullying of children with autism. While they did not find a gender difference, Libster et al. (37) found that children with greater social skills were bullied more than those with lower social skills. This is possibly the case due to individuals with high social skills are more likely to interact with their bullies, leading to bullying behaviour. Low self-esteem has been identified as a risk factor for bullying among autistic individuals (76). In another study, 722 teachers and 119 parents reported that older age and behavioural difficulties are associated with bullying victimisation of autistic children, but no significant difference was found in bullying rates by gender (77).

### The impact of bullying on the mental health of autistic individuals

Bullying has been found to have a profound impact on the mental health of individuals with autism, leading to a range of negative outcomes such as depression, low self-esteem, negative self-concept, loneliness, and anxiety (20, 78–80).

In a study involving 219 adolescents with autism and their parents conducted by Chou et al. (20), victims of bullying reported significantly higher levels of depression and anxiety than those who did not experience bullying. Similar findings were reported by Morton et al. (80). These findings emphasise the negative impact of bullying on the mental health of autistic people. In Ghanouni and Quirke (78), three autistic individuals reported having mental health issues, although it was not clear if this was due to bullying. Bullying was found to be the main factor in many negative outcomes depression, anxiety and suicidal ideation (81). For similar results, see also Mikami et al. (82) and Chang et al. (83). Secci et al. (84) reported on a case showing suicidal ideation, depression and being bullied, although it is not clear if these are interrelated. Bullied victims most likely will experience depression (1), anxiety (37) and suicidal thoughts (85). Using the nationally representative National Survey of Children’s Health (NSCH), Accardo et al. (86) found that anxiety and depression are coming bullied autistic children.

In addition, bullying victimisation in autism was found to lead to psychopathological disorders later in life (87), including the development of psychosis, which also impact mental health. Similar findings were reported by Stanyon et al. (88). Another recent study also found that bullying victimisation in autism leads to self-harm and isolation as well as lack of sense of belonging (89).

In their research Cassidy et al. (90), investigated the experiences of 50 autistic individuals who had been subjected to bullying; they reported a higher rate of bullying in comparison to their typically developing peers in addition to comorbid anxiety and depression. In one study, autistic students shared their struggle with social interactions which lead to significance levels of depression, anxiety, and anger due to bullying victimisation and mistreatment by their typically developing peers (77). According to many research studies, parents reported a significant connection between bullying and anxiety symptoms experience by their autistic children (91).

In context of young adults, McLeod et al. (92) found significant differences between autistic students and their non-autistic peers in terms of bullying, and physical and mental health. Parents also identified a strong association between bullying and social anxiety in their autistic children (44). These findings highlight the importance of understanding the specific challenges faced by autistic individuals during critical life transitions and the potential impact on their mental health. Additionally, the correlation between bullying and psychiatric comorbidity, including depression and emotional dysregulation, further emphasises the need for comprehensive support systems (38).

## Discussion

In sum, there are a multitude of studies on the prevalence of bullying of children with autism in different countries. Different studies reported different prevalence rates, ranging from 20% to 90%. It is not clear why there are major differences among the different studies, however, cultural and societal expectations and norms may be a key factor.

Prior met-analysis studies have also reported autistic individuals are likely to experience bullying compared to their neurotypical peers (93, 94), which agrees with our findings here. In their meta-analysis Park et al. (93), estimated the bullying victimisation among autistic individuals with 67% estimate of victimisation, 29% perpetration, and 14% perpetration-victimisation, which was much higher than neurotypical individuals. Specifically, 58% of verbal bullying, followed by 36% relational, then 30% physical bullying, and 15% cyber-victimisation. In another meta-analysis study, Maiano et al. (22) reported that autistic children experience higher rates of bullying when compared to their typically developing peers. According to a systematic review by Sterzing et al. (67), up to 63% of autistic individuals have experienced bullying at some point in their lives. A recent systematic review study found that bullying of children with autism leads to development of depression and anxiety, as found in over 40 studies (95).

Sreckovic et al. (21) reviewed 21 articles and found that autistic individuals are frequent victims of bullying. Bullying victimisation among autistic individuals is 2.4 times higher than their non-autistic peers and two times higher than other disabilities with autistic individuals experiencing more physical bullying and social exclusion (97). Similarly, Schroeder et al. (96) highlighted the need for a more comprehensive knowledge and response by shedding light on the widespread issue of bullying encounters experienced by autistic children. Several factors may account for the wide variation. First, methodological differences including variations in study design, sample size, and measurement tools can influence reported prevalence. Second, contextual and cultural factors may play a role, for example, studies conducted in Western countries often report higher rates than those in Asian or Middle Eastern contexts, potentially reflecting differences in awareness, stigma, or reporting practices, as low rates of bullying were reported in Indonesia (98). Third, population characteristics (e.g., age group, gender distribution, severity of autism characteristics, school placement in mainstream versus special education settings) are likely to shape the extent and type of bullying reported. Finally, definitions of bullying have not been consistent across studies, with some including only overt forms (physical and or verbal) and others also considering relational or cyberbullying, which can lead to substantial variability in prevalence estimates.

Autistic Children are estimated to experience 33% of verbal bullying, 50% of physical bullying and 31% of relational bullying (22).

An analysis of the risk factors for bullying of autistic people must consider the social context in which the bullying occurs, with much extant research focusing instead on the individual traits, often positioned as deficits, of the autistic person. Drawing on the social model of disability, the challenges experienced by autistic individuals are conceptualised not as a result of the individual’s traits or social behaviours, but rather as a result of the neurotypical barriers and attitudes that exist within the individual’s environment, which may in turn create the conditions for bullying to occur. We argue, in line with the social model of disability, that bullying occurs not because an autistic person has social deficits, but because social environments are commonly defined by often unspoken neurotypical rules of communication that are inaccessible to certain neurotypes. Thus it is critical to challenge societal norms that perpetuate the notion that conformity is the only acceptable behaviour and consider the broader social context within which bullying occurs.

According to the evidence, bullying has a significant impact on the mental health of autistic people. Addressing this issue requires comprehensive interventions that focus on both bullying prevention and providing appropriate support for individuals who have been victims of bullying. It is critical that society recognises and addresses the unique challenges that autistic people face in order to foster inclusive environments that promote their mental health and overall well-being. More research is needed to develop effective strategies and support systems that can mitigate the negative effects of bullying while also promoting the resilience and empowerment of autistic people. In summary, the impact of bullying on the mental health of autistic individuals necessitates a critical examination of the underlying societal factors and systemic biases. While interventions focusing on bullying prevention and support for victims are crucial, it is equally important to promote inclusive environments that challenge societal norms and biases, foster acceptance, empathy, and understanding. Future research should aim to develop comprehensive strategies that address the unique challenges faced by autistic individuals, while also promoting their resilience and empowerment. By recognising and addressing these complex factors, society can work towards creating a supportive and inclusive environment for autistic individuals.

### Implications for prevention and protection

To address the significant impact of bullying on the mental health and well-being of autistic people, effective prevention and protection strategies should be developed. A multifaceted approach involving educators, parents, policymakers, and the larger community is essential. By reviewing the literature, we found that there are different ways to prevent and protect autistic people from bullying, including education and awareness, creating inclusive environments, building resilience and self-advocacy skills, collaboration, mental health support, and support from speech-language pathologists. Below, we discuss each one of these methods in turn.

Education and Awareness: Increasing understanding and empathy among peers, educators, and the broader community by increasing education and awareness about autism and the specific challenges faced by autistic individuals (67). Additionally, education might also focus on identifying the ways in which societal practices might be exclusive rather than inclusive. Educators should be trained in recognising and responding to bullying behaviours, as well as creating inclusive environments and implementing evidence-based anti-bullying interventions (99). The perception of the educators about diversity and its value is critical in structuring a safe environment for autistic individuals (100). Such education should seek to spread awareness about human differences and neurodiversity with a focus on acceptance and increasing positive attitudes towards autism (75).

Creating Inclusive Environments: Many children strongly identify with their groups and exclude others based on group functioning, autism included. Social exclusion is fed by stigma discrimination, and fear of threat, and therefore contribute to bullying. To establish contact with members of other groups facilitate effective social inclusion and positive attitudes, since contact between those with different neuro-statuses occurs organically across life contexts, however, additional facilitated contact could be valuable, especially in school contexts (71). Thus, creating inclusive environments in schools and communities is critical for preventing and responding to bullying. Fostering an accepting culture, celebrating neurodiversity, and encouraging positive social interactions among all students can all help to create inclusive environments (29). Increased contact with peers with autism helps eliminate negative beliefs and lay the foundation for inclusive attitudes and better understanding of the condition (100). Opportunities for social skill development and empathy teaching can also improve inclusivity and support (101).

Building Resilience and Self-Advocacy Skills: Giving autistic people the tools they need to develop resilience and self-advocacy skills can help them navigate bullying situations more effectively (1). Building resilience requires teaching assertiveness skills, providing support networks, and promoting self-esteem and self-confidence (44). Encouraging open communication and putting in place reporting mechanisms can help autistic people seek help and protection (78). Nonetheless, it is also helpful to such skills training be directed towards those who are engaging in bullying to help them navigate and better understand the fear and sense of threat that may be driving their bullying behaviour (102).

Collaboration: Effective prevention and protection requires collaboration among schools, parents, and community organisations. Clear lines of communication, involving parents in anti-bullying initiatives, and fostering partnerships with autism support organisations can all help to improve collective efforts (22). Collaboration allows for a coordinated response to bullying incidents and comprehensive support for autistic people (46).

Policymakers should prioritise the development and implementation of comprehensive anti-bullying policies and legislation that specifically addresses the needs of autistic people. Prevention strategies, reporting mechanisms, disciplinary measures, and provisions for support and intervention services should all be included in these policies (22). Legislation should ensure that all people, including those on the autism spectrum, have the right to a safe and inclusive educational environment (20).

Mental Health Support: It is critical to provide autistic individuals who have been bullied with accessible and appropriate mental health support services (known as neuroaffirmative practice). School counsellors, psychologists, and mental health professionals should be trained to understand autistic people’s unique mental health needs and to provide targeted interventions such as therapy and support groups (38).

Support from Speech-language pathologists: In addition to the above, some also rely on speech-language pathologists to enhance social communication in children with autism who have suffered from bullying victimisation (103). It is argued that speech pathologists can help reduce bullying in children with autism (104). However, it has been pointed out in the literature that some speech-language pathologists lack knowledge of bullying issues children with autism face, while some indicated that they feel uncomfortable interfering in these issues as these are not at the core of their practice (105).

To summarise, preventing and protecting autistic people from bullying requires a multifaceted approach that includes education, inclusivity, resilience-building, collaboration, policy, mental health support, and ongoing research. We can reduce the vulnerability of autistic people to bullying and promote their overall well-being by implementing these strategies and creating a supportive environment.

### Limitations and future directions

While the current review highlights consistent evidence linking autism and elevated risk of bullying, it is important to acknowledge several limitations in the underlying literature. First, the majority of studies adopt a cross-sectional design, meaning that data were collected at a single point in time. Although this approach is useful for identifying associations between bullying, autism, and mental health, it restricts the ability to establish causality. For example, it remains unclear whether bullying directly precipitates poorer mental health outcomes for autistic individuals, or whether pre-existing vulnerabilities increase the likelihood of being bullied. Future research should therefore employ longitudinal designs that can capture changes over time and clarify developmental trajectories.

A second limitation is the possibility of publication bias, whereby studies reporting significant associations are more likely to be published, while null or non-significant findings are underrepresented. This imbalance can distort prevalence estimates and contribute to overgeneralisation. Along these lines, another limitation of our study is not we did not conduct risk of bias analysis. The reason for doing so is, we had very strict inclusion criteria as well as we wanted to include as many papers as possible in order to address the complex relationship between autism and bullying, which can be seen in the 5 categories of autism-bullying relationships discussed in the Results section above. However, there is a risk that some of our 74 studies found through our PRISMA search could have risks of confounding, selection of participants, missing data, measurement of the outcome, and/or selection of the reported results.

Third, cultural and contextual differences in measurement complicate interpretation. Studies vary in how bullying is defined (e.g., overt aggression versus inclusion of relational and cyberbullying), and these definitional inconsistencies contribute to the wide range of reported prevalence. Furthermore, differences in school systems, cultural attitudes toward disability, and willingness to disclose victimisation may shape both actual experiences and reported rates of bullying.

Taken together, these issues highlight important research gaps. There is a need for cross-cultural comparative studies that use standardised instruments to disentangle methodological effects from contextual influences. In addition, under-researched regions, including the Arab world, require more empirical attention. Finally, research should move beyond prevalence estimates to examine protective factors, intervention efficacy, and policy implementation, thereby generating actionable evidence to inform inclusive practice.

As for future directions, it has been reported that students who experience challenges in forming and maintaining friendships increase the possibilities of peer victimisation (106). However, future work should consider conducting this with autistic individuals.

Future research should also investigate demographic and cultural variables that may mediate bullying victimisation of individuals with autism, such as tight vs. loose cultures (107).

## Conclusion

In conclusion, the existing literature unequivocally demonstrates the profound impact of bullying on the well-being and mental health of autistic individuals. The implications for prevention and protection outlined in this review underscore the significance of various strategies, including education, creating inclusive environments, resilience-building, collaboration, policy development, mental health support, and ongoing research.

While it is crucial to address bullying prevention and provide appropriate support for victims, it is equally important to critically examine the societal factors that contribute to the perpetuation of bullying among autistic individuals. Stigmatising attitudes, lack of support, and exclusionary practices in society can perpetuate feelings of inadequacy, contribute to the development of low self-esteem, and further increase vulnerability to bullying (1). This highlights the need for inclusive attitudes, supportive environments, and challenging societal norms and biases that contribute to the vulnerability of autistic individuals.

Efforts to prevent and address bullying must involve multiple stakeholders, including educators, parents, policymakers, and the wider community. Education and awareness initiatives are crucial in fostering understanding and empathy, cultivating a culture that celebrates neurodiversity, and promoting positive social interactions. By actively promoting inclusive environments and providing opportunities for social skills development, schools and communities can create a supportive and accepting atmosphere that actively discourages bullying. Empowering autistic individuals with resilience and self-advocacy skills is pivotal in navigating bullying situations effectively. Building self-esteem and assertiveness, coupled with maintaining open lines of communication and establishing reporting mechanisms, can empower autistic individuals to seek help and protection when faced with bullying incidents.

Collaboration between schools, parents, and community organisations plays a key role in developing comprehensive prevention and protection strategies. Through collaborative efforts, stakeholders can coordinate responses to bullying incidents, implement evidence-based interventions, and establish support networks specifically designed for autistic individuals.

Policy and legislation must address the specific needs of autistic individuals, ensuring that anti-bullying policies encompass prevention strategies, effective reporting mechanisms, disciplinary measures, and provisions for support and intervention services. Legal frameworks should safeguard the rights of all individuals, including those on the autism spectrum, to a safe and inclusive educational environment.

Access to appropriate mental health support services is critical for autistic individuals who have experienced bullying. School counsellors, psychologists, and mental health professionals should receive specialised training to understand and address the unique mental health needs of autistic individuals, offering targeted interventions and support groups. Continued research is paramount to deepen our understanding of the dynamics of bullying among autistic individuals and to evaluate the effectiveness of prevention and protection strategies. Longitudinal studies can identify risk and protective factors, inform evidence-based interventions, and contribute to the development of policies and practices that promote the well-being of autistic individuals.

Importantly, more research is needed to better understand the dynamics of bullying among autistic individuals and to assess the efficacy of prevention and protection strategies. Longitudinal studies can identify risk and protective factors, as well as the long-term effects of bullying on mental health outcomes (1). This information can be used to inform evidence-based interventions and policy recommendations. Future research should strive to conceptualise the pathology of bullying in context to both perpetrators and the non-inclusive societal expectations that influence interpersonal behaviour across contexts, noting that autistic individuals can perpetrate bullying as well as experience it. Little research has addressed these issues, nor investigated how bullying occurs across the spectrum, including instances of bullying perpetrated by an autistic person. The nuances of bullying and neurodiversity could be further explored by examining differences in autistic-to-autistic person bullying in contrast to non-autistic-to-autistic person bullying.

Finally, just as it has been argued in the disability community that it is not someone’s inability to climb stairs that creates their experience of “dis-ability” but rather the presence of stairs in conjunction with a spinal injury, for example, that creates “dis-ability”, so too the bullying of autistic individuals must be conceptualised in context to dominant, non-inclusive social mores and not merely individual differences. Many of the challenges autistic individuals experience in social contexts are created when traits associated with autism collide with a social environment that expects individuals to adhere to neurotypical social standards. It is the coalescing of autistic traits with neurotypical social expectations that creates conditions ripe for bullying.

More inclusive social standards may be essential in reducing the challenges experienced by autistic individuals and may help to reduce rates of their bullying. Inclusive research and practice standards, alongside the implementation of inclusive prevention and protection protocols are critical to collective efforts to reduce the vulnerability experienced by autistic individuals and the fostering of an inclusive society that values and supports neurodiversity. Through these efforts, we can create safe and accepting environments that prioritise the well-being and uphold the dignity of all individuals on the autism condition.

Further, it is important to acknowledge certain methodological omissions in this review. In particular, no formal risk of bias assessment or evidence certainty evaluation was conducted on the included studies. While the synthesis provides valuable insights into the prevalence, types, and consequences of bullying among autistic individuals, the absence of these methodological steps limits the ability to fully assess the strength and reliability of the evidence base. Recognising this limitation is critical to ensure transparency and to allow readers to interpret the conclusions with appropriate caution. Future systematic reviews should incorporate structured risk of bias tools and certainty grading frameworks to strengthen methodological rigor and enhance confidence in the findings presented.

## Data Availability

The original contributions presented in the study are included in the article/**Supplementary Material**. Further inquiries can be directed to the corresponding author.

## Supplementary material

Supplementary Table 1 PRIMS Guidelines.

Supplementary Table 2 List of studies found in our PRIMA search.
