# Supplementary material for: Autism, bullying, and mental health: a comprehensive systematic review
Source: Front Psychiatry. 2025 Dec 5;16:1653663. doi: 10.3389/fpsyt.2025.1653663 (PMC12715608; doi:10.3389/fpsyt.2025.1653663)
Supplement: Supplementary Table 1 — PRIMS Guidelines. [file Table1.docx]

# Supplementary Material

Table S1: PRIMS Guidelines

| **Section and Topic** | **Item #** | **Checklist item** | **Location where item is reported** |
| --- | --- | --- | --- |
| **TITLE** | | |  |
| Title | 1 | Identify the report as a systematic review. | 1 |
| **ABSTRACT** | | |  |
| Abstract | 2 | See the PRISMA 2020 for Abstracts checklist. | 2 |
| **INTRODUCTION** | | |  |
| Rationale | 3 | Describe the rationale for the review in the context of existing knowledge. | 9 |
| Objectives | 4 | Provide an explicit statement of the objective(s) or question(s) the review addresses. | NA |
| **METHODS** | | |  |
| Eligibility criteria | 5 | Specify the inclusion and exclusion criteria for the review and how studies were grouped for the syntheses. | 9 |
| Information sources | 6 | Specify all databases, registers, websites, organisations, reference lists and other sources searched or consulted to identify studies. Specify the date when each source was last searched or consulted. | 10 |
| Search strategy | 7 | Present the full search strategies for all databases, registers and websites, including any filters and limits used. | 10 |
| Selection process | 8 | Specify the methods used to decide whether a study met the inclusion criteria of the review, including how many reviewers screened each record and each report retrieved, whether they worked independently, and if applicable, details of automation tools used in the process. | 9 |
| Data collection process | 9 | Specify the methods used to collect data from reports, including how many reviewers collected data from each report, whether they worked independently, any processes for obtaining or confirming data from study investigators, and if applicable, details of automation tools used in the process. | 9,10 |
| Data items | 10a | List and define all outcomes for which data were sought. Specify whether all results that were compatible with each outcome domain in each study were sought (e.g. for all measures, time points, analyses), and if not, the methods used to decide which results to collect. | NA |
|  | 10b | List and define all other variables for which data were sought (e.g. participant and intervention characteristics, funding sources). Describe any assumptions made about any missing or unclear information. | NA |
| Study risk of bias assessment | 11 | Specify the methods used to assess risk of bias in the included studies, including details of the tool(s) used, how many reviewers assessed each study and whether they worked independently, and if applicable, details of automation tools used in the process. | NA |
| Effect measures | 12 | Specify for each outcome the effect measure(s) (e.g. risk ratio, mean difference) used in the synthesis or presentation of results. | As this is a systematic review and not a meta analysis, given the diverse nature of studies included |
| Synthesis methods | 13a | Describe the processes used to decide which studies were eligible for each synthesis (e.g. tabulating the study intervention characteristics and comparing against the planned groups for each synthesis (item #5)). | 11,12 |
|  | 13b | Describe any methods required to prepare the data for presentation or synthesis, such as handling of missing summary statistics, or data conversions. | As this is a systematic review and not a meta analysis, given the diverse nature of studies included |
|  | 13c | Describe any methods used to tabulate or visually display results of individual studies and syntheses. | As this is a systematic review and not a meta analysis, given the diverse nature of studies included |
|  | 13d | Describe any methods used to synthesize results and provide a rationale for the choice(s). If meta-analysis was performed, describe the model(s), method(s) to identify the presence and extent of statistical heterogeneity, and software package(s) used. | 11,12 |
|  | 13e | Describe any methods used to explore possible causes of heterogeneity among study results (e.g. subgroup analysis, meta-regression). | Does not apply as this is a systematic review |
|  | 13f | Describe any sensitivity analyses conducted to assess robustness of the synthesized results. | Does not apply as this is a systematic review |
| Reporting bias assessment | 14 | Describe any methods used to assess risk of bias due to missing results in a synthesis (arising from reporting biases). | See Discussion for rationale |
| Certainty assessment | 15 | Describe any methods used to assess certainty (or confidence) in the body of evidence for an outcome. | See Discussion for rationale |
| **RESULTS** | | |  |
| Study selection | 16a | Describe the results of the search and selection process, from the number of records identified in the search to the number of studies included in the review, ideally using a flow diagram. | 20-23 |
|  | 16b | Cite studies that might appear to meet the inclusion criteria, but which were excluded, and explain why they were excluded. | 20 |
| Study characteristics | 17 | Cite each included study and present its characteristics. | 12-19 |
| Risk of bias in studies | 18 | Present assessments of risk of bias for each included study. | NA |
| Results of individual studies | 19 | For all outcomes, present, for each study: (a) summary statistics for each group (where appropriate) and (b) an effect estimate and its precision (e.g. confidence/credible interval), ideally using structured tables or plots. | Does not apply as this is a systematic review |
| Results of syntheses | 20a | For each synthesis, briefly summarise the characteristics and risk of bias among contributing studies. | NA |
|  | 20b | Present results of all statistical syntheses conducted. If meta-analysis was done, present for each the summary estimate and its precision (e.g. confidence/credible interval) and measures of statistical heterogeneity. If comparing groups, describe the direction of the effect. | Does not apply as this is a systematic review |
|  | 20c | Present results of all investigations of possible causes of heterogeneity among study results. | Does not apply as this is a systematic review |
|  | 20d | Present results of all sensitivity analyses conducted to assess the robustness of the synthesized results. | Does not apply as this is a systematic review |
| Reporting biases | 21 | Present assessments of risk of bias due to missing results (arising from reporting biases) for each synthesis assessed. | NA |
| Certainty of evidence | 22 | Present assessments of certainty (or confidence) in the body of evidence for each outcome assessed. | Does not apply as this is a systematic review |
| **DISCUSSION** | | |  |
| Discussion | 23a | Provide a general interpretation of the results in the context of other evidence. | 20 |
|  | 23b | Discuss any limitations of the evidence included in the review. | 26 |
|  | 23c | Discuss any limitations of the review processes used. | 26 |
|  | 23d | Discuss implications of the results for practice, policy, and future research. | 27 |
| **OTHER INFORMATION** | | |  |
| Registration and protocol | 24a | Provide registration information for the review, including register name and registration number, or state that the review was not registered. | Review was not registered |
|  | 24b | Indicate where the review protocol can be accessed, or state that a protocol was not prepared. | NA |
|  | 24c | Describe and explain any amendments to information provided at registration or in the protocol. | NA |
| Support | 25 | Describe sources of financial or non-financial support for the review, and the role of the funders or sponsors in the review. | NA |
| Competing interests | 26 | Declare any competing interests of review authors. | NA |
| Availability of data, code and other materials | 27 | Report which of the following are publicly available and where they can be found: template data collection forms; data extracted from included studies; data used for all analyses; analytic code; any other materials used in the review. | NA |

Table S2: List of studies found in our PRIMA search.

| **Study** | **Study focus** | **Study details** | **Main findings** |
| --- | --- | --- | --- |
| Gkatsa & Antoniou, 2024 | Prevalence of bullying | This study investigated bullying in high functioning autistic individuals. | Around 20% of the sample experienced bullying more than twice a week. Around 40% experienced bullying once a week |
| Libster et al., 2022 | Prevalence of bullying  & risk factors underlying bullying | The study focused on whether restricted repetitive  behaviors, and autism severity impact bullying victimisation. | Austistic individuals experience bullying victimisation more than non-austistic individuals.  Autistic people with higher social skills are bullied more than ones with lower social skills. |
| Zablotsky et al. 2023 | Impact of bullying on mental health | The study focused on risk factors underlying being a bullying victim or a bully | Bullying increases internatising symptoms and emotional dysregulation |
| Twyman et al. (2010) | Prevalence of bullying | They used Reynolds’ Bully-Victimisation Scale to investigate bulying | Austistic individuals experience bullying more than non-austistic individuals |
| Humphrey & Hebron, 2015 | Prevalence of bullying | They compared bullying in autistic individuals in comparison to individuals with disabilities | Austistic individuals experience bullying more than individuals with disabilities. |
| van Roekel et al. 2010. | Prevalence of bullying | They investigated theory of mind in relation to bullying. | Prevalence of bullying in austistic individuals is between 6 and 46%. |
| Lung et al., 2019 | Prevalence of bullying | They investigated prevalence of bullying in autism in Taiwan | Prevalence of bullying in austistic individuals is over 60%. |
| Ball & Zhu, 2023 | Prevalence of bullying | They used parents’ reports to investiagte bullying in autistic and non-austistic individuals | Austistic individuals experience bullying more than non-austistic individuals |
| Hwang et al. 2018 | Prevalence of bullying | They investigated the impact of comorbid psychopathology in autism in relation to bulying | Austistic individuals experience bullying more than non-austistic individuals but controlling for comborbid psychopahtology removes this effect. |
| Carter et al., 2009 | Prevalence of bullying | How common is bullying in Asperger’s syndrome? | 65% of Asperger individuals face bullying |
| Rowley et al., 2012 | Prevalence of bullying | They investigated the relationship of autism to number of friends. | Austistic individuals experience bullying and low levels of friendships compared to students with special needs. |
| Humphrey & Symes, 2010 | Prevalence of bullying | They compared bullying in students wit autism and also students with special needs | Austistic students experienced more bullying than students with special needs |
| Batten et al., 2006 | Prevalence of bullying | Study investigated prevalence of bullying in autistm | Prevalence of bullying in autism is around 40% |
| Little, 2001 | Prevalence of bullying | Prevalence of bullying in autism | Prevalence of bullying in autism is over 70% |
| Wainscot, 2008 | Prevalence of bullying | Prevalence of bullying in autism | Prevalence of bullying in autism is over 70% |
| Hebron et al., 2017 | Prevalence of bullying | Parental engagement and its relation to bullying | Parental engagement to protect against bullying |
| Little, 2002 | Prevalence of bullying | How common is bullying in Asperger’s individuals | it is over 90% in this population. |
| Paul et al. 2028 | Prevalence of bullying | This study investigates bullying in schools in France | Over 90% of austistic students face bullying. |
| Pryke-Hobbes et al. (2023) | Prevalence of bullying & impact of bullying on mental health | Bullying in autistic adults. | Austistic adults experience bullying at work. |
| Gotby et al., 2018 | Prevalence of bullying | Autism and coercive sexual victimisation | Autistic women, who are three times more likely to experience coercive sexual victimisation than non-autistic women. |
| Almarzouq et al. (2024) | Bullying and autism in non-Western contexts | Bullying in schools in Kuwait | Bullying leads to social isolation, decreased participation in school activities and friendship. |
| Al-Saleh (2019) | Bullying and autism in non-Western contexts | Bullying in Saudi Arabia | Bullying impacts inclusive education |
| Alatawi, 2023 | Bullying and autism in non-Western contexts | Bullying in Saudi Arabia | Autistic individusla face sexual, social, verbal, and physical bullying in different regions in Saudi Arabia |
| Daghustani (2024), | Bullying and autism in non-Western contexts | Bullying in Bahrain | Verbal bullying is more common than social bullying, which is more common than physical bullying |
| Alzaidi (2017) | Bullying and autism in non-Western contexts | Bullying in Saudi Arabia | Teacher presence protects autistic kids from bullying |
| Binhayyan (2017) | Bullying and autism in non-Western contexts | Bullying in Saudi Arabia | Austistic kids experienced more bullying than non-austistic kids |
| Daghustani & MacKenzie, 2021 | Bullying and autism in non-Western contexts | Bullying in Bahrain | Autistic kids experience  verbal and physical bullying in mainstream schools |
| Al-Makahleh et al.,2024 | Bullying and autism in non-Western contexts | Bullying in Jordan | Physical and verbal bullying is common among austistic children |
| Khamis et al.,2024 | Bullying and autism in non-Western contexts | Mothers’ report of bullying of their autistic children in Jordan | Physical and verbal bullying is common among austistic children in Jordan |
| Bitsika et al., 2022 | Prevalence and impact of of bullying | Bullying and school refusal | Bullying increases school refusal |
| Furukawa et al., 2023 | Prevalence and impact of of bullying | Bullying and school refusal | Bullying increases school refusal and hospitalisation |
| Ochi et al., 2020 | Prevalence and impact of of bullying | Bullying and school refusal | Bullying increases school refusal in boys and girls. |
| Oliveira et al., 2023 | Types of bullying | Autobiographica reprots of the types of bullying | Austistic individuals experience verbal and physical bullying |
| Saigh & Bagadood, 2022 | Types of bullying | Interviewing mothers regarding types of bullying their children face | Austistic individuals experience social, verbal and physical bullying |
| Adams et al., 2016 | Types of bullying | Investigating type of bullying autistic individuals face | Austistic individuals experience different types of victimisation including aggression. |
| Campbell et al., 2017 | Types of bullying | Investigating type of bullying autistic individuals face | Compared to typically developing individuals, austistic individuals experience verbal, social, and physical bullying |
| Kloosterman et al. (2013) | Types of bullying | Investigating type of bullying autistic individuals face | Austistic individuals experience social bullying more than non-autitic individuals. |
| Blake et al., 2012 | Prevalence of bullying | Bullying in autism vs. individuals with disabilities | Austistic individuals experience bullying more than non-autistic individuals |
| Zeedyk et al. 2014 | Prevalence and types of bullying | Which types of byllying experiences more in autistic individuals? | Austistic individuals experience verbal and physical bullying more than non-autistic individuals |
| Cappadocia et al. (2012) | Risk factors underlying bullying | Which factors increase bullying in autism? | Communication problems and poor understanding of autism are risk factors |
| De-la-Iglesia, & Olivar, (2015 | Risk factors underlying bullying | What are the risk factors for bullying |  |
| Saigh and Bagadood (2022), | Types of bullying | Interview with mothers on types of bullying | Austic students face different kinds of bullying including verbal and physical types. |
| Triantafyllopoulou et al., 2022 | Prevalence of bullying | Cyberbullying prevalence in adults | autistic adults are at a higher risk of being victim of cyberbullying than non-autistic adults |
| Pearson et al., 2023 | Risk factors underlying bullying | What risk factors lead to discrimination in autism? | negative beliefs held by others result in discrimination |
| Chu et al., 2019 | Risk factors underlying bullying | What risk factors lead to discrimination in autism? | Psychosical problems increase risk of bullying in autistic individuals. |
| Hsiao et al., 2022 | Risk factors underlying bullying | What risk factors lead to bullying in autism? | Lower social skills increase risk of bullying in autistic individuals. |
| Liu et al., 2022 | Risk factors underlying bullying | What risk factors lead to harrassement in autism? | Impulsivity increased the risk of harrssement in autism |
| Montes et al., 2007 | Risk factors underlying bullying | What risk factors lead to harrassement in autism? | Co-morbid ADHD increases the likeloood of bullying in autism |
| Junttila et al., 2024 | Risk factors underlying bullying | What risk factors lead to bullying in autism? | Loneliness increases risk of bullying in autism. |
| Turnock et al., 2022 | Risk factors underlying bullying | What risk factors lead to bullying in autism? | Social impairment, such as lack of eye contact, is a risk factor for bullying. |
| Hsiao et al., 2022 | Risk factors underlying bullying | What risk factors lead to bullying in autism? | Diagnosis with clinical disorders increase risk for bullying in autism. |
| Cook et al. 2020 | Risk factors underlying bullying | What risk factors lead to bullying in autism? | Positive interaction with non-austistic individuals decrease bullying. |
| Wang &Yokota, 2024 | Risk factors underlying bullying | Which risk factors lead to bullying in autism? | Low self-esteem increases bullying in autism. |
| Hebron & Humphrey, 2014 | Risk factors underlying bullying | What risk factors lead to bullying in autism? | Behavioural difficulties are associated with bullying. |
| Morales-Hidalgo et al., 2024 | Risk factors underlying bullying | What risk factors lead to bullying in autism and others with subthreshhold autistic traits? | Bullying was associated with severity of restrictive, repetitive, behavioural and emotional problems. |
| Chou et al., 2020 | Impact of bullying on mental health | How does bullying impact mental health in autism? | Bullying leads to an increases in depression and anxiety in autism. |
| Ghanouni & Quirke, 2022; | Impact of bullying on mental health | Coping strategies in autism. | Three austistic adults stated having mental illnesses. |
| Morton et al., 2024 | Impact of bullying on mental health | How does bullying impact mental health in autism? | Bullying leads to an increases in depression and anxiety in autism. |
| Bal et al., 2022 | Impact of bullying on mental health | How does bullying impact mental health in autism? | Bullying leads to suicide ideation in autistic individuals. |
| Secci et al., 2023 | Impact of bullying on mental health | How does bullying impact mental health in autism? | Case study of an individual reporting depression and and suicide ideation and being bullies. However, links among these were not clear in this study. |
| Ferrigno et al., 2022 | Impact of bullying on mental health | How does bullying impact mental health in autism? | Bullying in autism leads to psychosis, which is related to mental health issues |
| Stanyon et al., 2022 | Impact of bullying on mental health | How does bullying impact mental health in autism in Japan? | Bullying in autism leads to psychosis, which is related to mental health issues |
| Eroglu et al., 2019 | Types of bullying | What types of bullying victimisation do autistic individuals face in Turkey? | Austic students face different kinds of bullying including emotional and verbal types. |
| Haegele & Maher, 2022 | Impact of bullying on mental health | How do autistic students feel during physical education classes? | Autistic students are bullied often, which leads to social isolation and self-harm as well as lack of sense of belonging. |
| Mayes et al., 2013 | Impact of bullying on mental health | How does bullying impact mental health in autism? | Austistic children show an increased level of suicide ideation and also report high levels of bullying. |
| Cassidy et al. (2014) | Impact of bullying on mental health | Relationship between prevalence of bullying and depression and anxiety | Bullying leads to an increases in depression and anxiety in autism. |
| Weiss et al., 2015 | Impact of bullying on mental health | Relationship between bullying and anxiety | Bullying leads to an increases in anxiety in autism. |
| Rodriguez et al., 2021 | Risk factors underlying bullying | What risk factors lead to bullying in autism? | Prior mental health problems, severity of autism are related to an increase in bullying. |
| McLeod et al. (2019) | Impact of bullying on mental health | Relationship between bullying and mental health in autistic adults | Bullying leads to an increases in mental health problems in autistic adults. |
| Accardo et al., 2024 | Impact of bullying on mental health | Relationship between bullying and depression and anxiety in autism | Austistic kids who are bullied suffer from depression and anxiety more than others who are not bullied. |
| Deniz et al., 2024 | Prevalence of bullying | This study investigates sibling bullying | Austistic children are also bullied by their siblings, which impacts their self esteem. |
| Ashburner et al., 2019 | Impact of bullying on mental health | Parental reports of bullying in their autistic children. | Anxiety and depression occur due to bullying in autism. |
| Mikami et al., 2009 | Impact of bullying on mental health | Investigation of bullying and suicide relationship | Suicide is common among autistic individula who are bullied. |
| Chang et al., 2024 | Impact of bullying on mental health | Does bullying lead to suidide in autism | Bullying victimisation was a factor underlying suicide in autism. |
